# Dewetting Process of Silver Thin Films and Its Application on Percolative Pressure Sensors with High Sensitivity

**DOI:** 10.3390/polym15010180

**Published:** 2022-12-30

**Authors:** Chia-Yu Cho, Jui-Chen Chang, Min-Xian Cai, Pei-Ting Lin, Yao-Joe Yang

**Affiliations:** Department of Mechanical Engineering, National Taiwan University, Taipei 10617, Taiwan

**Keywords:** silver thin film, nanoparticle arrays, percolation, dewetting process, pressure sensor

## Abstract

This work reports on an innovative dewetting process of silver thin films to realize percolative nanoparticle arrays (NPAs) and demonstrates its application on highly sensitive pressure sensors. The dewetting process, which is a simple and promising technique, synthesizes NPAs by breaking the as-deposited metal film into randomly distributed islands. The NPA properties, such as the mean particle size and the spacing between adjacent particles, can be easily tailored by controlling the dewetting temperature, as well as the as-deposited metal-film thickness. The fabricated NPAs were employed to develop gauge pressure sensors with high sensitivity. The proposed sensor consists of a sealed reference-pressure cavity, a polyimide (PI) membrane patterned with an interdigital electrode pair (IEP), and a silver NPA deposited on the IEP and the PI membrane. The operational principle of the device is based on the NPA percolation effect with deformation-dependence. The fabricated sensors exhibit rapid responses and excellent linearity at around 1 atm. The maximum sensitivity is about 0.1 kPa^−1^. The advantages of the proposed devices include ultrahigh sensitivity, a reduced thermal disturbance, and a decreased power consumption. A practical application of this pressure sensor with high resolution was demonstrated by using it to measure the relative floor height of a building.

## 1. Introduction

Miniaturized precision pressure sensors are vital for numerous applications in various fields. In recent years, the development of high-resolution pressure sensors for measuring ambient pressure has received great attention because of the rapid progress of wearable systems and mobile devices [1,2,3,4,5,6,7]. Various types of nanomaterials, including two-dimensional layers [8,9,10], nanotubes [11,12,13], nanofibers [14,15,16], and nanoparticles (NPs) [17,18,19], were proposed as the key materials for these pressure-sensing devices. In general, the sensing principle is based on the electron tunneling transport between closely spaced NPs, which gives rise to a sharp change in electronic conductance induced by the structure deformation [20]. However, the sensitivities of these devices are usually moderate, and some of the polymer-based approaches suffer from a high hysteresis effect [21]. Therefore, in recent years, percolative nanoparticle arrays (NPAs), which exhibit high sensitivity, as well as strain tolerance, have been proposed as piezoresistive transducing elements for ultrasensitive pressure sensors. In general, a percolative NPA is formed by depositing NPs arranged in a configuration so that the spacing between adjacent NPs is sufficiently small that the electron transport between neighboring NPs is dominated by the electrons tunneling and hopping across energy barriers.

Magnetron sputtering systems with gas aggregation cluster sources (GASs) are one of the most popular tools for the fabrication of a wide variety of metallic NPAs. For example, a device with strain-sensing elements with a high sensitivity realized by the GAS sputtering technique was proposed [22]. The device sensitivity is about ten times higher than that of devices with traditional metallic films. In addition, Du et al. [23] employed a similar sputtering process to realize a strain sensing device based on chromium NPAs with a sensitivity this is about one hundred times higher than metallic- or semiconductor-based strain sensors. In [20], an ultrahigh sensitive piezoresistive pressure sensor with percolative dense metal NPs deposited on a flexible polyethylene terephthalate membrane was proposed. The device exhibits a very high resolution of about 0.5 Pa.

These approaches employed a GAS to realize high-quality percolative NPAs for excellent strain-sensing devices. However, GAS sputtering systems are more complex than typical sputtering tools. In addition, it is well known that a GAS is quite inefficient in terms of target material utilization because the redeposition on the target surface is uncontrollable [24]. In this work, we propose a simple technique to fabricate percolative NPAs for a high-resolution pressure sensor by using a standard sputtering tool with an innovative dewetting process [25,26]. The pressure sensor comprises of an NPA, an interdigital electrode pair (IEP) on a flexible membrane, and a substrate with a cavity. The metal NPA is fabricated by dewetting a thin metal film by using a standard sputter with a special annealing step. The dewetting process, which is driven by the energy minimization of all surfaces and interfaces in an as-deposited film, is a simple and promising technique for realizing ordered NPAs. In addition, by controlling the annealing temperature, as well as the as-deposited film thickness, the NPA properties, such as the mean particle size and the spacing between the adjacent particles, can be tailored [27]. This dewetting process is quite simple and repeatable [28]. Additionally, the size of the NPs and the distance between NPs may be controlled independently [29]. The sensing principle of the NPA is based on the percolation effect with deformation-dependence. The advantages of the proposed devices include ultrahigh sensitivity, reduced thermal noises, and a reduced power consumption. In summary, the aim of this work is to develop an innovative dewetting process of silver thin films by using a standard sputtering tool to realize percolative nanoparticle arrays (NPAs) and demonstrate its application on highly sensitive pressure sensors.

## 2. Materials and Methods

### 2.1. Design Strategy of Percolative Sensing Principle

**Figure 1:**
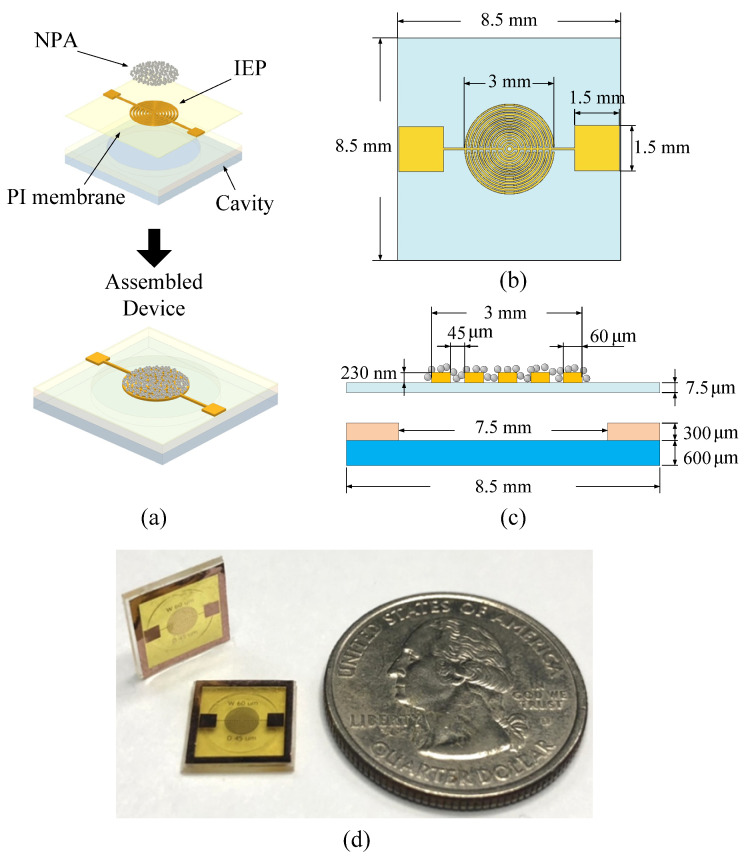
(**a**) Schematic of the proposed pressure sensor. (**b**) The top view and (**c**) the cross-sectional view of the device. (**d**) The picture of the fabricated devices.

This sub-section described the sensing principle of percolative NPAs and realized the proposed dewetting process of the silver thin film. Figure 1a shows the schematic and the exploded view of the proposed pressure sensor. The device consists of a sealed reference-pressure cavity and a polyimide (PI) membrane patterned with an IEP. In addition, an array of randomly distributed silver NPs deposited on the IEP and the PI membrane serves as the key pressure-sensing element using the percolation phenomenon. Figure 1b,c are the top view and the cross-sectional view of the device, respectively. The dimensions of the device are also indicated in the sub-figure. The thickness of the IEP is 230 nm, and the width of each IEP finger is 60 µm. The gap between IEP fingers is 45 µm.

**Figure 2:**
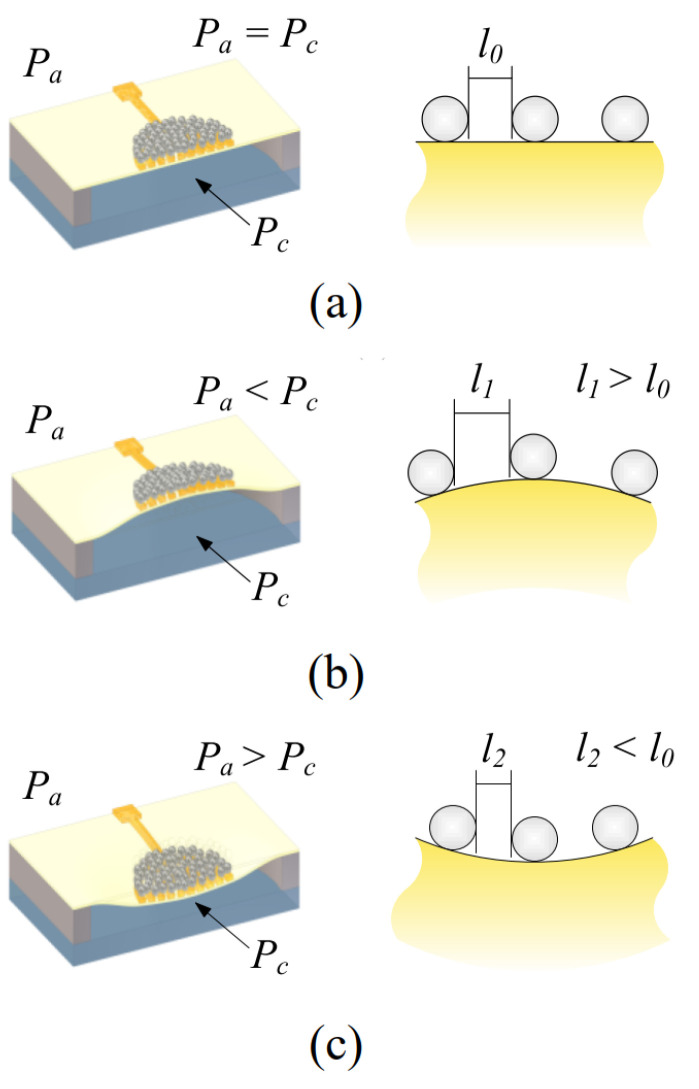
The device operational principle for three different conditions: (**a**) *P*_a_ = *P*_c_, (**b**) *P*_a_ < *P*_c_, and (**c**) *P*_a_ > *P*_c_.

The device’s operational principle is shown in Figure 2, where *P*_a_ is the ambient pressure. *P*_c_ is the cavity pressure created during the cavity-sealing process and serves as the reference pressure. The figure on the right side is a schematic view illustrating the distance between silver NPs. As *P*_a_ is the same as *P*_c_ (Figure 2a), the PI membrane is undeformed, and the average distance between NPs is $l_{0}$. As *P*_a_ is smaller than *P*_c_ (Figure 2b), the membrane deforms upward, which induces a tensile strain around the central region of the top surface of the membrane [20]. Consequently, the average distance between adjacent NPs increases ($l_{1}>l_{0}$), resulting in the destruction of the percolation pathways, which results in an increase in the NPA sheet resistance [22,30,31,32]. Figure 2c is the schematic illustrating the behavior when *P*_a_ is larger than *P*_c_. The mean distance between silver NPs decreases ($l_{2}<l_{0}$). Thus, the number of percolative pathways in the whole sensing element increases, which in turn decreases the NPA sheet resistance. Hence, the resistance change in the NPA, which can easily be measured with the IEP patterned on the PI membrane, reflects the pressure difference between *P*_a_ and *P*_c_. Based on the model presented in [30], the ratio of the electrical conductivity (resistance) of the thin films can be described by the following equation:

(1) $$\frac{R}{R_{0}}=\frac{\sigma_{0}}{\sigma }=\exp[-\beta (l_{0}-l)]\exp\left[\frac{0.5e^{2}}{4\pi \varepsilon_{r}\varepsilon_{0}kT}\left(\frac{1}{r+l_{0}}-\frac{1}{r+l}\right)\right]$$

where e is the electron charge (1.6 × 10^−19^ C), $\varepsilon_{0}$ is the permittivity in air (8.854 × 10^−12^ F/m), $\varepsilon_{r}$ is the relative permittivity in air (1), k is the Boltzmann’s constant (1.38 × 10^−23^ J/K), T is the ambient temperature (300 K), *β* is the electron coupling term (about 4 nm^−1^ [32]), r represents the particle radius, and $l_{0}$ represents the undeformed interparticle average spacing (nm), and l represents the deformed interparticle average spacing (nm).

Equation (1) can be further simplified as Equation (2) since the second exponential term is very close to unity [31].

(2) $$\frac{R}{R_{0}}=\frac{\sigma_{0}}{\sigma }=\exp[-\beta (l_{0}-l)]=\exp[\beta (\Delta l)]$$

where $\Delta l=l-l_{0}$.

### 2.2. Device Fabrication and Dewetting Process

The main structure of the proposed device was fabricated using micromachining techniques. The silver NPA on the electrode pairs was realized by applying the dewetting process on a silver thin film [33]. In general, an as-deposited metallic thin film is not stable, and therefore tends to agglomerate to form arrays of isolated islands when heated. Note that both the dewetting process and the surface tension phenomenon can be described by the Young–Laplace equation [25]. Therefore, forming tiny islands (nanoparticles) in the dewetting phenomenon can be analogous to forming liquid droplets caused by surface tension, which is achieved by the energy minimization of all surfaces. In addition, the dewetting process could happen well below the metal melting temperature.

**Figure 3:**
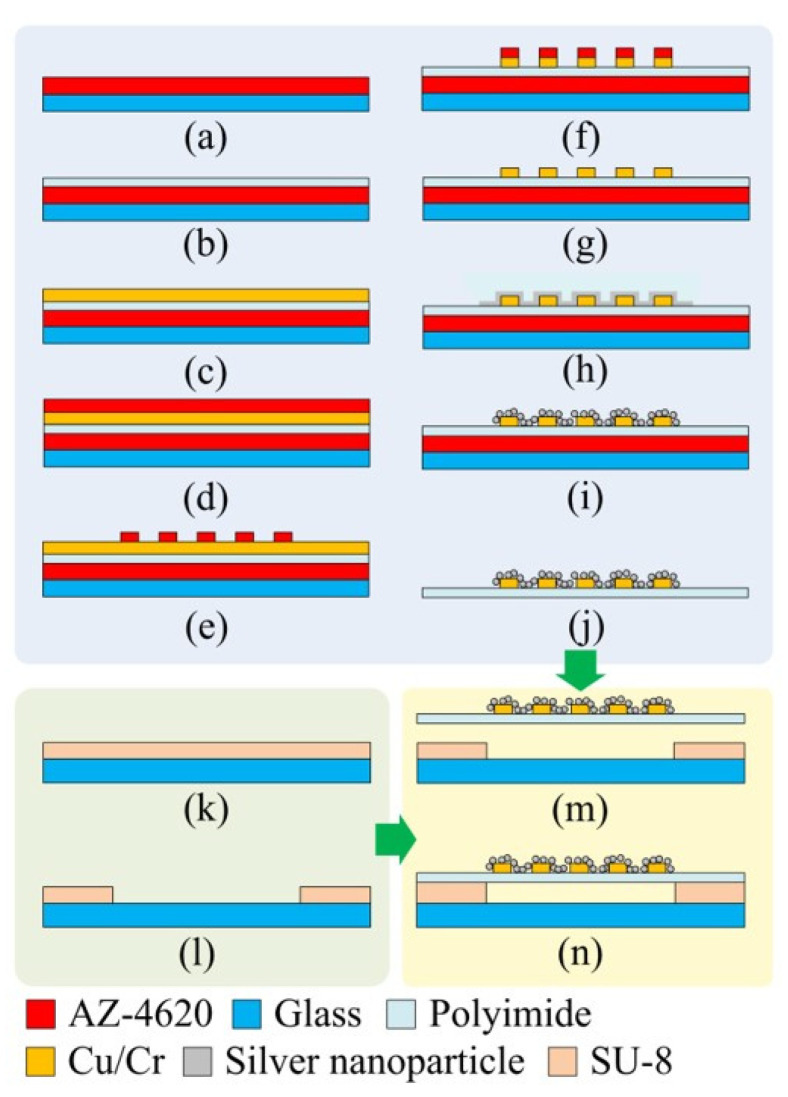
The fabrication process of the proposed device.

Figure 3 illustrates the micromachining process to fabricate the proposed pressure sensing device. The IEP layer was patterned onto the PI film (Kapton^®^ KJ, DuPont, Taipei, Taiwan) using standard micromachining techniques (Figure 3a–g). First, a layer of an AZ-P4620 photoresist film (10-µm) was spin-coated onto a glass wafer (Figure 3a). The photoresist serves as a sacrificial layer. Then, a PI layer was deposited onto the sacrificial layer (Figure 3b). A 30-nm chromium (Cr) layer, which served as an adhesion layer for copper (Cu) layer deposition, was then deposited by a sputtering process. Subsequently, a 300-nm Cu layer was sputtered onto the Cr layer (Figure 3c). Then, another layer of AZ-P4620 photoresist was spin-coated (Figure 3d). This layer was patterned as the IEP etching mask (Figure 3e). The IEP was formed by a wet etching process (Figure 3f) using a copper etchant (CE-100, Transene Co., Danvers, MA, USA) and a chrome etchant (CR-7, Transene Co., Danvers, MA, USA). Then, the etching mask (AZ-P4620 photoresist) was removed by immersing the substrate in acetone (Figure 3g). Subsequently, a silver thin film of 6 nm in thickness was deposited onto the IEP using a sputter [34] (Figure 3h). The deposition rate was 3.5 Å·s^−1^. The whole area of the IEP was covered by the deposited silver film. Then, by using the dewetting process, silver NPs were created on top of the substrate (Figure 3i). The dewetting process is in fact an annealing process of the silver thin film at 200 °C for 60 min [35,36], and is performed in the chamber of the sputter without breaking the vacuum. Note that, according to our experimental results, the PI membrane starts to wrinkle as the annealing temperature for dewetting exceeds 230 °C. Once the PI membrane wrinkles, it is considered to be damaged. After the dewetting process, the membrane with the IEP was separated from the glass substrate by dissolving the first AZ-P4620 photoresist film (the sacrificial layer) using acetone, as shown in Figure 3j. Finally, the device was assembled by mounting the fabricated PI membrane onto a glass substrate with a SU-8 cavity (Figure 3m,n) and heating it above the glass transition temperature of SU-8 (SU-8 2050, MicroChem Corporation, Westborough, MA, USA). Note that the SU-8 cavity was formed by patterning a SU-8 layer using a standard lithography process (Figure 3k,l). Figure 1d shows the picture of the fabricated devices.

**Figure 4:**
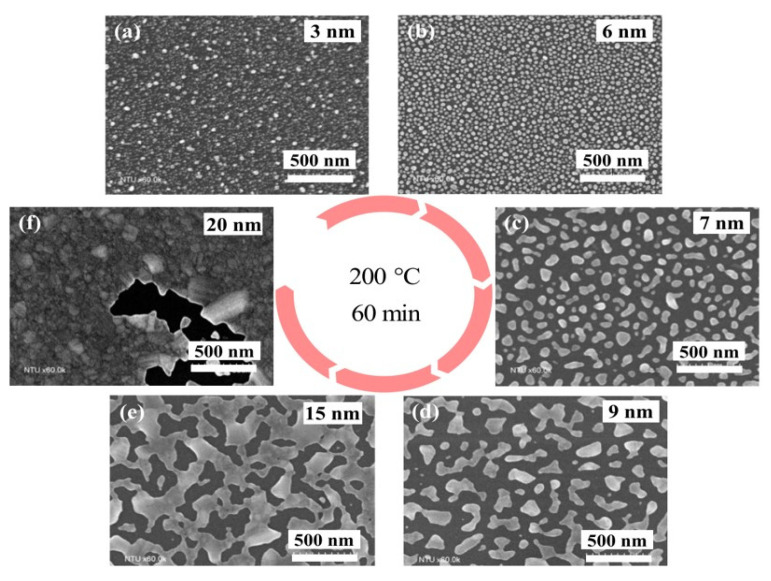
The SEM images of the dewetted results of the silver films with an original thickness of (**a**) 3 nm, (**b**) 6 nm, (**c**) 7 nm, (**d**) 9 nm, (**e**) 15 nm, and (**f**) 20 nm.

Figure 4 shows a series of scanning electron microscope (SEM) (FESEM, S-4800, Hitachi, Ltd., Chiyoda, Tokyo, Japan) images illustrating the dewetting results with different thicknesses of as-deposited silver films. Note that the deposition rate was measured by a thin film deposition monitor (SQM-160, INFICON^®®^, Bad Ragaz, Switzerland) that uses a quartz crystal sensor. The thickness resolution of the thin film deposition monitor is 0.037 Å, and the maximum error is 7%. Due to dewetting, the as-deposited films continuously break into randomly distributed islands. It was observed that relatively thin silver films (i.e., 3-nm and 6-nm films, as shown in Figure 4a,b) result in discrete silver-isolated islands (NPs) with almost round shapes, while relatively thick silver films (i.e., 7-nm, 9-nm, and 15-nm films, as shown in Figure 4c–e) form islands with elongated (irregular) shapes or connected islands. This is because the driving force for dewetting decreases as the original thickness of the silver film increases. In addition, the dewetting phenomenon is not obvious (or does not occur) for the film whose original thickness is relatively thick (i.e., 20-nm film, as shown in Figure 4f).

**Figure 5:**
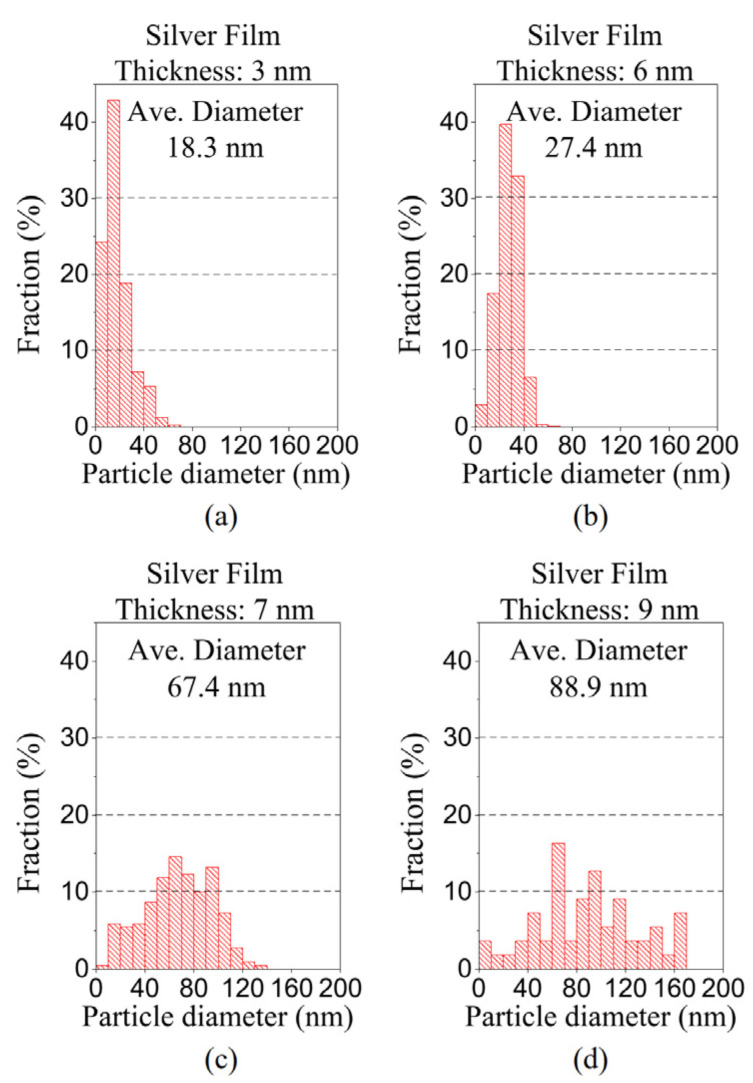
Particle distribution of the dewetted NPAs for the silver films with an original thickness of (**a**) 3 nm, (**b**) 6 nm, (**c**) 7 nm, and (**d**) 9 nm.

Figure 5 shows the diameter distributions of the particles (i.e., isolated islands or irregular/connected islands) after dewetting. These results indicate that, as the thickness of the silver film (before dewetting) increases, the average diameter of the particles (after dewetting) increases, while the particle diameter’s standard deviation also increases significantly. Obviously, a 6-nm-thick silver film yields an NPA with the highest particle density and the best distributional uniformity. We use an image processing program ImageJ (National Institute of Health, Bethesda, MD, USA) to estimate particle diameters, as well as analyze the diameter distribution of particles from high resolution SEM images.

**Figure 6:**
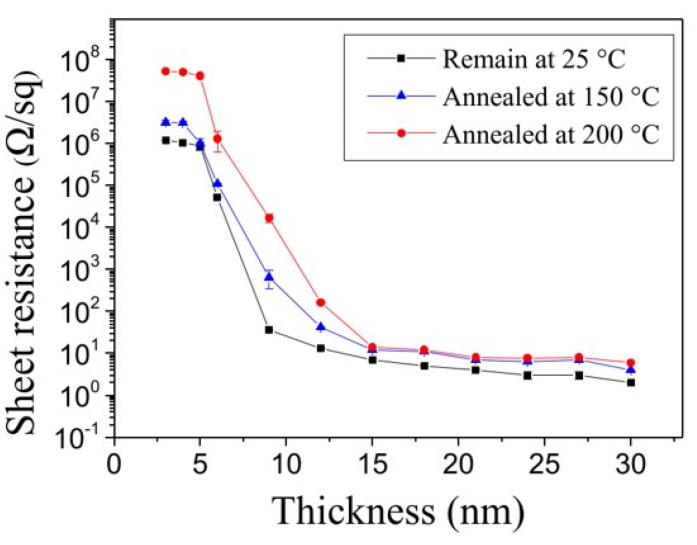
Relationship of the sheet resistance of dewetted silver films vs. the original thicknesses of the silver films prior to dewetting at different annealing temperatures.

Figure 6 shows the sheet resistance of dewetted silver films vs. the original thicknesses of the silver films prior to dewetting. Each curve presents the results with the same annealing temperature. Note that the sheet resistance was measured by using a four-point probe system (KSR-4, Everbeing Int’l Corp., Hsinchu, Taiwan) with a source meter (Keithley 2400, Keithley Instruments, Inc., Cleveland, OH, USA). For each curve (i.e., at a specific annealing temperature), the sheet resistance displays a dramatic drop of six orders of magnitude when the silver film thickness increases. Further increases in the film thickness result in a gradual reduction in the decrease in sheet resistance. This behavior of a sharp resistance change can be described by the SEM images in Figure 4. As shown in Figure 4, the silver films with relatively thin original thicknesses (i.e., the films with original thicknesses of 3 nm, 6 nm, and 7 nm) result in uniformly isolated nanoparticles after the dewetting process. Therefore, the sheet resistances of these nanoparticle arrays are quite high, as indicated in Figure 6. Furthermore, the silver films with relatively thick original thicknesses (i.e., the films with original thicknesses of 9-nm, 15-nm, and larger) form islands with elongated and connected particles after dewetting. Since a large portion of the dewetted particles are electrically connected, the sheet resistances of these dewetted films are very small. This phenomenon aligns well with the percolation effect [37] and can be employed to implement highly sensitive strain sensing elements in response to the tunneling effect due to the distance change between adjacent isolated particles caused by deformation.

## 3. Results and Discussion

**Figure 7:**
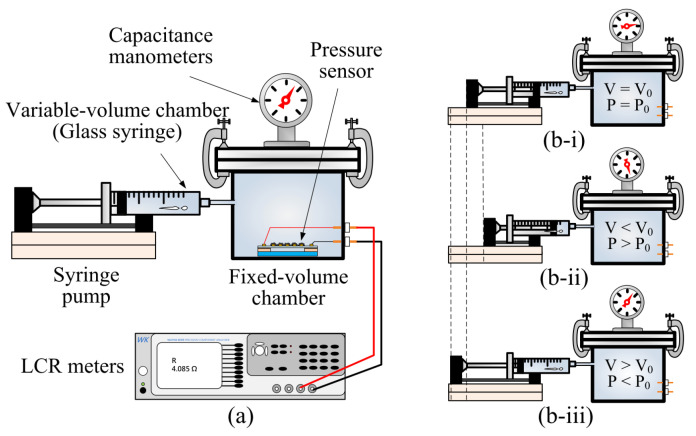
(**a**) Schematic diagram of experimental setup for pressure measurement. (**b**) The schematic of the internal pressure changes of the chamber system when the volume of the variable-volume chamber (syringe pump) (**b-i**) does not change, (**b-ii**) decreases, and (**b-iii**) increases.

The measurement setup for characterizing the fabricated sensors is shown in Figure 7a. The setup consists of a fixed-volume chamber (2500 cc) and a variable-volume chamber (about 120 cc) that is actually a glass syringe installed on a computer-controlled syringe pump. The variable-volume chamber is connected to a fixed-volume chamber, and the whole system is completely sealed. Therefore, the total internal volume of the chamber system is the sum of the volumes of the fixed-volume chamber and the variable-volume chamber. In addition, four electrical feed-throughs are installed on the fixed-volume chamber for connecting the sensors with external instruments. A capacitance manometer (626C, MKS Instruments, Andover, MA, USA) is also connected to the fixed-volume chamber to measure the internal pressure of the chamber. Figure 7b shows the schematic of how to change the internal pressure of the chamber. As the syringe pump slowly pushes (pulls) the plunger of the syringe, the total volume of the system decreases (increases), and thus the internal pressure of the system increases (decreases). The fabricated sensor is connected electrically to an LCR meter (6440A, Wayne Kerr Electronics Co., Woburn, MA, USA) that is located outside of the fixed-volume chamber through a vacuum-compatible feed-through.

**Figure 8:**
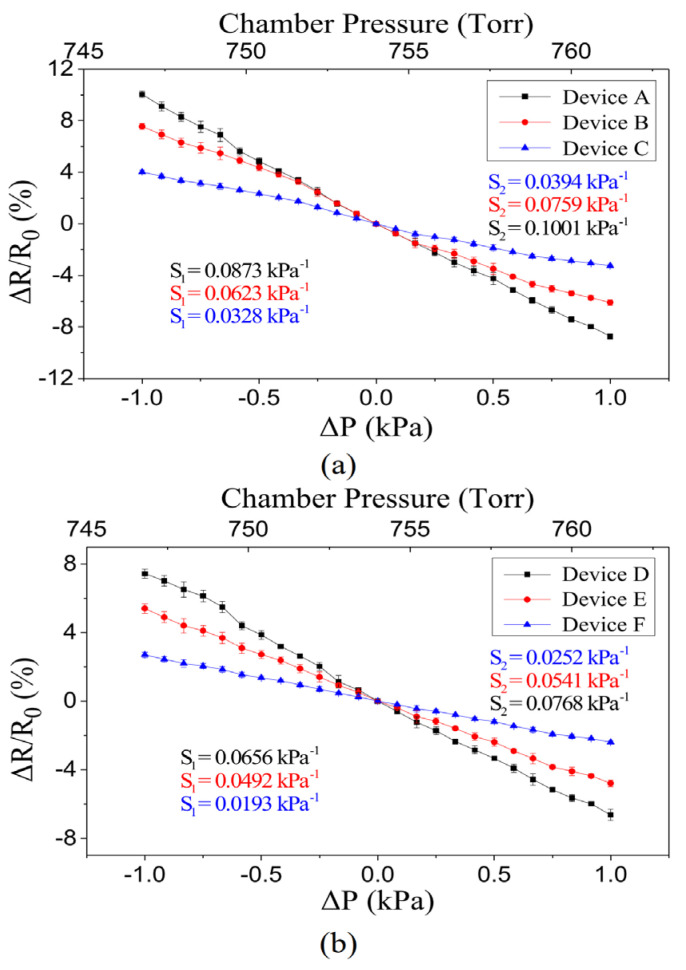
(**a**) Comparison of devices with different film thickness in 7.5 mm cavity. (**b**) Comparison of devices with different film thickness in 6.0 mm cavity.

Figure 8a,b show the relationship between the measured resistance change vs. the pressure variation for the fabricated pressure-sensing devices with different design parameters listed in Table 1. More than 10 samples were manufactured for each device configuration (i.e., devices A, B, C, D, E and F). The cavity diameters of the devices in Figure 8a,b are 7.5 mm and 6.0 mm, respectively. In addition, the three curves shown in each figure result from devices with different PI membrane thicknesses. Each data point on these curves is the average result of 10 measurements. The error bars of each data point indicate the measured maximum and minimum values. The figures show that, as the ambient pressure is greater than the cavity reference pressure (i.e., ΔP>0), a compressive strain is induced on the top surface of the membrane, resulting in a decrease in resistance. Similarly, a negative pressure difference (i.e., ΔP<0), which induces a tensile strain, causes an increase in resistance. The slopes of these curves correspond to the sensor sensitivities. In addition, each curve consists of two lines with slightly different slopes, S_1_ and S_2_. The slope S_1_ is associated with the condition shown in Figure 2b, and the slope S_2_ is associated with Figure 2c. These measured results also indicate that the sensor with a thinner membrane exhibits a higher sensitivity because substantially more membrane deformation can be induced at the same applied pressure. In addition, devices with larger cavity diameters (when the size of the IEP is the same) are more sensitive due to the smaller effective stiffness of their membranes [38]. A brief modeling of this behavior can be found in the Supplementary Materials. Among these devices, the maximum sensitivity is about 0.1 kPa^−1^.

We can further estimate the variations of the average spacing (i.e., Δl) using Equation (2). According to Figure 8a, device A has the largest value of the maximum resistance variation (i.e., 10%). Therefore, we take device A as an example to evaluate the associated Δl. Note that $\Delta l=\Delta l_{up}=l_{1}-l_{0}$ if the membrane deforms upward, and $\Delta l=\Delta l_{down}=l_{2}-l_{0}$ if the membrane deforms downward. Based on Equation (2), $\Delta l_{up}$ is 0.024 nm, and $\Delta l_{down}$ is −0.026 nm. In addition, by using ImageJ to analyze the SEM picture shown in Figure 4b (the NPA employed to implement the proposed sensors),$\;l_{0}$ is estimated as 9.36 nm. Therefore, $l_{1}$ is approximately 9.384 nm and $l_{2}$ is 9.334 nm for device A.

**Figure 9:**
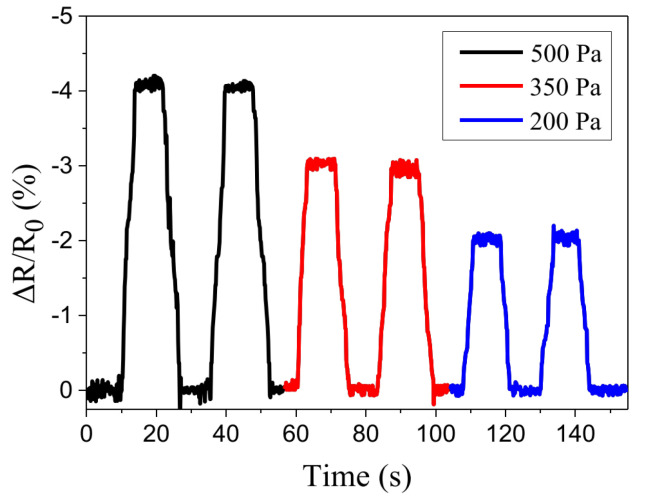
The response of the sensor under periodic loading and unloading cycles.

**Figure 10:**
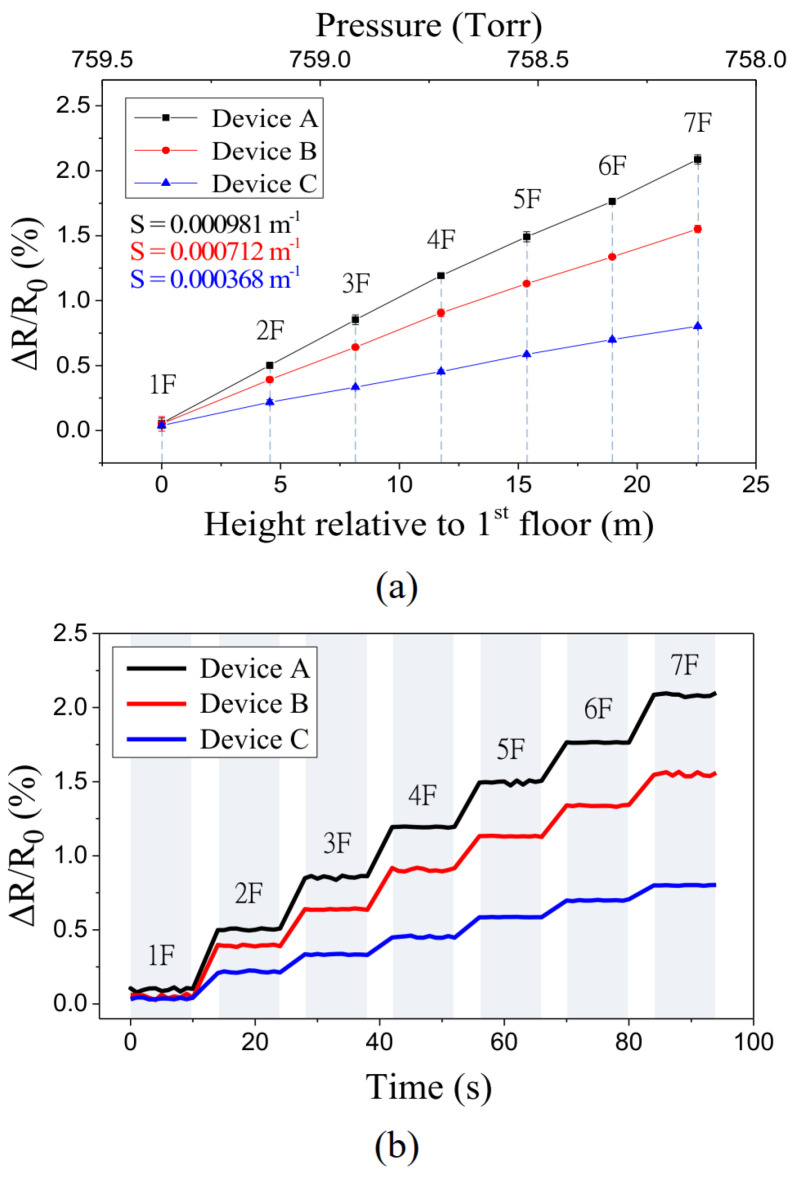
(**a**) Resistance changes in response to the variation in the floor elevation. (**b**) Transient response of the sensor when moving with an elevator from the 1st floor to the 7th floor.

Figure 9 shows the transient responses of device A (shown in Table 1) under cyclic pressure loadings. For each cycle, the NPA’s resistance rapidly changes in line with the applied pressure. A decrease in NPA resistance corresponds to a decrease in the mean distance between NPAs as the applied pressure increases. The return of the NPA’s resistance to its original level corresponds to the release of the applied pressure. One practical application of the fabricated sensors with ultrahigh resolution was demonstrated by using it to measure the altitude of the floors of a building. Figure 10a shows the sensor responses to the relative height of each floor. During the measurement period, the sensors move with an elevator that starts on the 1st floor, makes a stop on each floor, and finally reaches the 7th floor. Each data point is the average value of 10 measurements. The ambient pressure for each floor was also measured using a capacitance manometer (626C, MKS Instruments, Andover, MA, USA). Again, the results indicate that the sensitivity was higher for the device with a thinner membrane. Figure 10b shows the transient responses of the sensors as the elevator moves up and makes a stop on each floor for about 10 s. Each curve was measured during an elevator ride (from the 1st floor to the 7th floor). The transient curves closely follow the motion of the elevator.

## 4. Conclusions 

In summary, we report on an innovative dewetting process of silver thin films to realize percolative nanoparticle arrays (NPAs). In addition, highly sensitive pressure sensors were also realized using the NPAs. The experimental results show that a sharp resistance change caused by the percolation effect of the silver thin film can be utilized to implement highly sensitive sensing devices. A series of SEM images illustrating the dewetting behaviors at different conditions were demonstrated. The characterization of the fabricated sensors with different dimensions was presented. The devices exhibit good linear responses at around 1 atm, with a maximum sensitivity of about 0.1 kPa^−1^. The measured transient responses of the devices under different cyclic pressure loadings were quite repeatable without signal recession. It was also demonstrated that the proposed sensor is capable of measuring the relative floor height of a building due to its excellent sensitivity.

## Figures and Tables

**Table 1:** Dimensions of the fabricated sensors.

	PI Membrane Thickness	Cavity Diameter
**Device A**	7.5 µm	7.5 mm
**Device B**	12.5 µm	7.5 mm
**Device C**	25 µm	7.5 mm
**Device D**	7.5 µm	6.0 mm
**Device E**	12.5 µm	6.0 mm
**Device F**	25 µm	6.0 mm

## Data Availability

Data available on request.

## Supplementary Materials

The following supporting information can be downloaded at: https://www.mdpi.com/article/10.3390/polym15010180/s1, the analytical model of the central deflection for a circular membrane with clamped edge under a uniformly distributed load.
