# Supplementary material for: Dewetting Process of Silver Thin Films and Its Application on Percolative Pressure Sensors with High Sensitivity
Source: Polymers (Basel). 2022 Dec 30;15(1):180. doi: 10.3390/polym15010180 (PMC9823326; doi:10.3390/polym15010180)
Supplement: Supplementary file 1 [file polymers-15-00180-s001.zip › polymers-2009878-supplementary.pdf]

Supplementary Materials

Dewetting Process of Silver Thin Films and Its Application on Percolative Pressure Sensors with High Sensitivity

Chia-Yu Cho, Jui-Chen Chang, Min-Xian Cai, Pei-Ting Lin and Yao-Joe Yang *

The analytical model of the central deflection for a circular membrane with clamped edge under a uniformly distributed load [38] is shown in Equation S1. This analytical model can be used to approximately estimate the force-deformation relationship of the membrane of the proposed sensors. The schematic of the membrane is shown in Figure S1.

$$d = \frac{pr^4}{64D} \quad \text{(S1)}$$

where d is the central deflection of the membrane, D is the flexural rigidity, r is the membrane radius, and p is the uniformly distributed load.

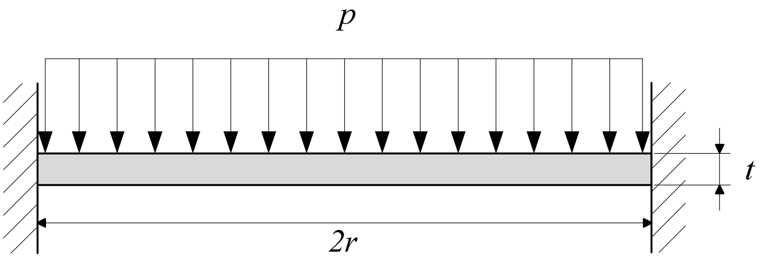

Figure S1. The cross-sectional schematic of a circular membrane with clamped edge under a uniformly distributed load.

According to Equation S1, the central deflection is proportional to r^4. Therefore, the average strain of the membrane is proportional to r^3 (i.e., d/r). Since the sensor response is proportional to the strain, the sensor response is also proportional to r^3. Therefore, under the same distributed load p, the device with a larger membrane diameter gives larger sensor response than the device with a smaller membrane diameter. Consequently, the device with a larger membrane diameter possesses a larger sensitivity. This brief modeling will be added in the supplementary material.

References

[38] S. Timoshenko and S. Woinowsky-Krieger, Theory of plates and shells. McGraw-hill New York, **1959**; pp. 51-58.
